# Effect of action-based cognitive remediation on cognition and neural activity in bipolar disorder: study protocol for a randomized controlled trial

**DOI:** 10.1186/s13063-018-2860-8

**Published:** 2018-09-12

**Authors:** Caroline V. Ott, Maj Vinberg, Christopher R. Bowie, Ellen Margrethe Christensen, Gitte M. Knudsen, Lars V. Kessing, Kamilla W. Miskowiak

**Affiliations:** 1 Copenhagen Affective Disorder Research Center (CADIC), Psychiatric Centre Copenhagen, Copenhagen University Hospital, Rigshospitalet, Copenhagen, Denmark; 2 Department of Psychology, University of Copenhagen, Copenhagen, Denmark; 3 Psychology Department, Queen’s University, Kingston, ON Canada; 4 Neurobiology Research Unit and Center for Experimental Medicine Neuropharmacology, Rigshospitalet, Copenhagen, Denmark; 5 Faculty of Health and Medical Sciences, University of Copenhagen, Copenhagen, Denmark

**Keywords:** Biomarker, Bipolar disorder, Cognition, Cognitive impairment, Cognitive remediation, Functional magnetic resonance imaging, Pro-cognitive effect

## Abstract

**Background:**

Cognitive impairment is present in bipolar disorder (BD) during the acute and remitted phases and hampers functional recovery. However, there is currently no clinically available treatment with direct and lasting effects on cognitive impairment in BD. We will examine the effect of a novel form of cognitive remediation, action-based cognitive remediation (ABCR), on cognitive impairment in patients with BD, and explore the neural substrates of potential treatment efficacy on cognition.

**Methods/design:**

The trial has a randomized, controlled, parallel-group design. In total, 58 patients with BD in full or partial remission aged 18–55 years with objective cognitive impairment will be recruited. Participants are randomized to 10 weeks of ABCR or a control group. Assessments encompassing neuropsychological testing and mood ratings, and questionnaires on subjective cognitive complaints, psychosocial functioning, and quality of life are carried out at baseline, after 2 weeks of treatment, after the end of treatment, and at a six-month-follow-up after treatment completion. Functional magnetic resonance imaging scans are performed at baseline and 2 weeks into treatment. The primary outcome is a cognitive composite score spanning verbal memory, attention, and executive function. Two complete data sets for 52 patients will provide a power of 80% to detect a clinically relevant between-group difference on the primary outcome. Behavioral data will be analyzed using mixed models in SPSS while MRI data will be analyzed with the FMRIB Expert Analysis Tool (FEAT). Early treatment-related changes in neural activity from baseline to week 2 will be investigated for the dorsal prefrontal cortex and hippocampus as the regions of interest and with an exploratory whole-brain analysis.

**Discussion:**

The results will provide insight into whether ABCR has beneficial effects on cognition and functioning in remitted patients with BD. The results will also provide insight into early changes in neural activity associated with improvement of cognition, which can aid future treatment development.

**Trial registration:**

Clinicaltrials.gov, NCT03295305. Registered on 26 September 2017.

**Electronic supplementary material:**

The online version of this article (10.1186/s13063-018-2860-8) contains supplementary material, which is available to authorized users.

## Background

Persistent moderate to severe cognitive impairment across several cognitive domains is seen in 30–70% of patients with bipolar disorder (BD) during periods of remission [1–3]. The cognitive impairment is directly associated with reduced functional capacity and poor occupational outcome [4, 5], with the latter being the greatest economic burden of BD [6]. Despite growing evidence for negative individual and societal consequences of cognitive impairment associated with BD, there is currently no available treatments with direct and lasting effects on cognition [7].

The search for effective treatments for cognitive impairment in BD is adversely related to the lack of a brain-based biomarker for cognitive improvement [8, 9]. In particular, the development of new treatments targeting the central nervous system typically rely on pre-clinical studies, which provide poor prediction of treatment effects in clinical trials [10]. Consequently, clinical trials investigating the pro-cognitive effects of candidate cognition treatments have produced overall disappointing or only preliminary results [7, 11]. However, evidence from a few small open-label, non-controlled studies suggests that cognitive remediation (CR) may have pro-cognitive effects in patients with BD [12, 13], and recent findings from a larger randomized clinical trial of 70 h of computerized CR in remitted patients with BD type I showed improvements on processing speed, visual learning, and memory and a cognitive composite measure [14].

We have previously conducted the first randomized, controlled clinical trial investigating the effect of a 12-week CR program in partially remitted patients with BD [15]. The treatment showed no effect on objective cognitive measures (primary outcome), although some aspects of subjective cognition improved [15]. This lack of efficacy could reflect a type 2 error, as it was not verified whether the patients had objectively measured cognitive impairment at enrolment and post hoc assessments revealed no objective impairment (in the group as a whole) in the targeted cognition domain (verbal memory), sustained attention, or executive function [15]. As accumulating evidence indicates that there is only a weak association between subjective cognitive complaints and objective cognitive impairment in patients with BD [16, 17], potential cognitive benefits could have been masked by ceiling effects. However, it is also conceivable that the CR treatment was not intense enough and relied too heavily on *compensatory strategies* rather than *intensive training* of cognitive skills. A new form of CR, action-based cognitive remediation (ABCR), aims to optimize traditional CR to promote cognitive flexibility and to transfer skills acquired during treatment sessions to patients’ everyday lives. It involves individual goal setting, an intense training program combining computerized training with practical in-session activities, and cognitively challenging tasks between sessions, and has shown promising effects [18]. Specifically, Bowie et al. [18] compared ABCR (*N* = 24) to traditional CR (*N* = 26) in a patient group with severe mental illness, including patients with BD. While both treatments improved cognition, ABCR had a greater effect on functional capacity than traditional CR [18]. This converges with a meta-analysis on CR trials in schizophrenia showing that the combination of CR and skills training had larger effects on patients’ functional capacity than CR alone [19]. The adaptation of ABCR to patients with BD may, therefore, not only improve cognitive skills but also increase patients’ functional capacity, with benefits for quality of life and societal costs.

Patients with BD in remission display aberrant (most consistently hypo-) activity in areas including the dorsolateral prefrontal cortex (dlPFC) and the ventrolateral prefrontal cortex during cognitive control tasks like working memory and strategic encoding [20, 21]. Changes in neural activity in the dlPFC have been observed in interventions demonstrating possible pro-cognitive effects in psychiatric disorders. In particular, a recent meta-analysis assessing the changes in neural activity following CR treatment in schizophrenia found increased activity in the lateral and medial prefrontal cortex to be the most robust indicator of treatment-associated cognitive improvement [22]. Our group has demonstrated changes in neural activity in dorsal and dorsomedial prefrontal areas during working memory and learning tasks following 8 weeks of weekly high-dose erythropoietin (EPO) vs. saline infusions in patients with affective disorders [23, 24]. These findings indicate that changes in the neural activity in the dorsal prefrontal cortex may be a possible biomarker for pro-cognitive effects of interventions targeting cognition.

### Aims and hypotheses

This study aims to assess the effect of ABCR vs. a control treatment on cognitive improvement in (i) BD patients in full or partial remission and to (ii) assess early neural changes indicative of potential treatment benefits on cognition.

We hypothesize (i) that ABCR vs. a control treatment has a beneficial effect on cognition in patients with BD in full or partial remission (the primary hypothesis of the study). We hypothesize (ii) that this treatment-associated improvement of cognition translates into better functional capacity at the 6-months follow-up assessment (secondary outcome). For the *exploratory* analysis of the neuronal underpinnings of these treatment effects, we hypothesize that ABCR will produce an early change in neural activity in the dorsal prefrontal cortex during working memory and strategic memory encoding in the direction of the prefrontal activity levels seen in a healthy control group (i.e., partial normalization) and this activity will correlate with ABCR-associated improvements in cognitive function.

## Methods/design

### Study design and participants

**Fig. 1:**
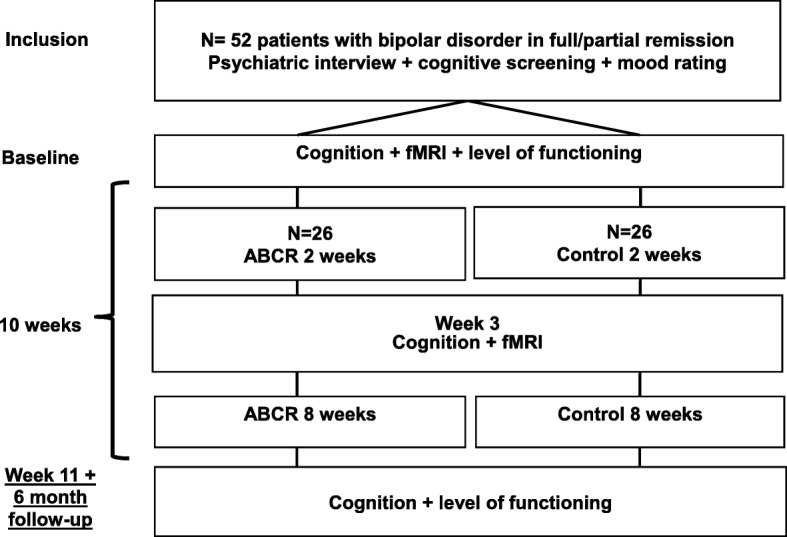
Flow diagram

See Fig. 1 for a flow diagram of the trial. The project has a randomized, controlled, outcome-assessor-blind, parallel-group design. The trial will include outpatients with BD in full or partial remission (score ≤ 14 on the Hamilton Depression Rating Scale [25] and Young Mania Rating Scale [26], respectively). No criteria for the duration of symptom stability is applied due to the feasibility of this group-based intervention trial, which requires starting groups three times per year. However, current mood symptoms and patients’ retrospective period of symptom stability will be recorded. Recruitment will be carried out through the ongoing Bipolar Illness Onset study [27], the Copenhagen Affective Disorder Clinic (Psychiatric Centre Copenhagen, Rigshospitalet), other mental health centers, consultant psychiatrists in the Capital Region of Denmark, and through advertisements on relevant websites.

Eligible participants must be between 18 and 55 years, be fluent in Danish, and present with *objective* cognitive impairment corresponding with a total score below the cutoff or scores below the cutoff on a minimum of two out of the five subtests (verbal learning test – immediate, working memory test, verbal fluency test, verbal learning test – delayed, and processing speed test) on the Screen for Cognitive Impairment in Psychiatry—Danish version (SCIP-D) [17, 28]. Patients are eligible if they have an ICD-10 diagnosis of BD (types I and II) confirmed using the Schedules for Clinical Assessment in Neuropsychiatry interview [29]. Their daily use of benzodiazepines will be tapered to a maximum dose equivalent to ≤22.5 mg oxazepam or ≤7.5 mg diazepam per day (cutoffs for doses with limited cognitive side effects). Other than that, patients are requested to avoid significant changes to dose and type of any medication prior to or during the study if possible. Any changes will be recorded at treatment completion and at the 6-month follow-up.

Exclusion criteria are current drug or substance abuse (up to 3 months prior to inclusion), previous serious head trauma, neurological illness, schizophrenia or schizoaffective disorder, dyslexia, claustrophobia, having a pacemaker or other metal implants inside the body, and electroconvulsive therapy in the 3 months prior to inclusion. Female participants are not included if they are pregnant. All participants must provide written informed consent, which includes consent to collection of biological material and for data to be used in ancillary studies. See Additional file 1 for a trial protocol checklist.

### Procedure

Upon their first visit to the Copenhagen Affective Disorder Research Center (CADIC), participants are informed about the project and provide written informed consent after which they undergo an eligibility assessment. The written informed consent will be obtained by the first author. Upon inclusion, participants are randomized with stratification for gender and age (< or ≥ 35 years) to either 10 weeks of ABCR (active treatment) or patient group (control treatment). When 4–6 participants have been randomized to a group, baseline assessments will be carried out in the week prior to the first group session. Deterioration in terms of clinical symptoms at the baseline assessment is recorded, but participants are not excluded if their symptoms worsen. The baseline assessment is completed over two days, 1–3 days apart for practical reasons and to avoid attrition.

On day 1, participants’ mood is rated with the Hamilton Depression Rating Scale and Young Mania Rating Scale, followed by a functional magnetic resonance imaging (fMRI) scan encompassing spatial and verbal working memory N-back tasks, a picture encoding task, a resting state, and a structural scan. On day 2, participants attend the research center in the morning for a fasting blood test. A comprehensive neuropsychological test battery is administered by a treatment-blinded outcome assessor. Participants fill in questionnaires concerning subjective cognitive complaints, psychosocial functioning, and quality of life. Functional capacity is assessed using a clinician-rated interview and a performance-based assessment. Finally, sleep quantity and quality in the past 3 days is assessed.

After 2 weeks of ABCR or control treatment, the fMRI, neuropsychological testing, and an assessment of mood and subjective cognition are repeated to assess whether early task-relevant neural activity changes—prior to improvement on the behavioral measures of cognition—correlate with and predict subsequent cognitive improvement after treatment completion. The neuropsychological assessments and questionnaires, as well as assessments of functional capacity and quality of life, are repeated within 2 weeks after treatment completion (primary outcome assessment time), and 6 months after treatment completion. An intermediate clinical mood rating is performed during week 6.

### Setting

The ABCR treatment and control treatment will take place at the outpatient clinic, CADIC, Psychiatric Centre Copenhagen, Rigshospitalet. Outcome assessments are performed at CADIC, Psychiatric Centre Copenhagen, Department O, Rigshospitalet.

#### Action-based cognitive remediation

ABCR is a manual CR program developed by Professor Christopher Bowie, Psychology Department, Queen’s University, Kingston, Ontario, Canada. It is traditionally carried out on groups of 4–6 participants with two therapists. The program covers the following cognitive domains: meta-cognition, verbal and visual working memory, memory, attention, and executive functions (organization, shifting attention, and planning). The program is carried out twice a week with each session lasting 2 hours. The program duration is 10 weeks, accompanied by daily computer training at home and homework assignments consisting of cognitively challenging everyday tasks (e.g. organizing stacks of documents, reading the newspaper, and making a budget). The computer program, the Danish version of HappyNeuron Pro (http://www.happyneuronpro.com), is administered on tablets and includes 28 tasks targeting processing speed, selective attention, working memory, verbal and visual learning, reasoning, and problem-solving. The program has 30 difficulty levels, and participants require an 80% success rate to advance to a more challenging level. Prior to the first ABCR session, participants receive an individual goal setting session, including identification of cognitive strengths and weaknesses based on the screening carried out during the eligibility assessment.

The ABCR program begins with an orientation session about the purpose and structure of the treatment and provides an opportunity for participants to state their personal goals to the group. Each session consists of a short presentation of the theme of the day followed by related computer activities and a joint discussion of strategies. Practical everyday-like activities (e.g. planning a meal and scheduling appointments) are then role-played keeping in mind the theme of the day and recently discussed strategies. The everyday-like activities are a main part of the sessions, and are performed using props such as food items, planners, city and amusement park maps etc. to increase the ecological validity of the activities. Each session ends with a discussion of how the content is related to each participant’s individual goal, and by identifying cognitively challenging everyday tasks for the participants to carry out between sessions. For four sessions within the program, the usual structure is replaced by computer training interleaved by 20-min individual goal setting sessions. Treatment completion is defined as 80% attendance and individual catch-up sessions are offered for missed sessions, if logistically possible. Attendance and time spent on the computer exercises between sessions will be recorded.

#### Control treatment

The control treatment is a weekly 1-h conversation group for 10 weeks. The sessions are conducted to control for the therapeutic effects of group treatment and are designed to *avoid* any training of cognitive abilities, but to remain meaningful for participants. Patients discuss their experiences of suffering from BD. There is no set structure in the groups, as relevant themes are suggested by the group leaders but ultimately decided by the participants. Group leaders primarily have a mediating function and serve to reinforce the time limit. A previous study showed that fewer participants dropped out of a group with a similar structure than in a psychoeducation group, which indicates general satisfaction with this group format [30].

### Treatment retention

All participants will receive feedback on the results of their neuropsychological results once they have completed the 6-month follow-up assessment. Additionally, participants randomized to the control group will be offered access to HappyNeuron Pro (www.happyneuronpro.com) following the 6-month follow-up-assessment. Participants who are working will be offered compensation of 100 Danish crowns an hour for 10 h of fMRI and neuropsychological assessments. Travel expenses for public transportation will be reimbursed for all participants.

### Randomization and blinding

Pharma Consulting Group carried out block randomization for each group stratified by gender and age (< or ≥ 35 years). Participants are randomized using a 1:1 allocation ratio. The study is outcome-assessor blind, and the randomization list and allocation envelopes are sealed in opaque envelopes and will be kept in a locked filing cabinet to prevent unblinding. The randomization list will not be opened during the trial enrolment phase or while the data analyses have not been completed. Allocation is carried out by the therapist, who will open the envelopes consecutively within each stratum following each eligibility assessment. Participants will be instructed not to disclose any information concerning their treatment allocation during assessments. Under no circumstances will the allocation be revealed to the outcome assessors.

### Outcome assessments

**Fig. 2:**
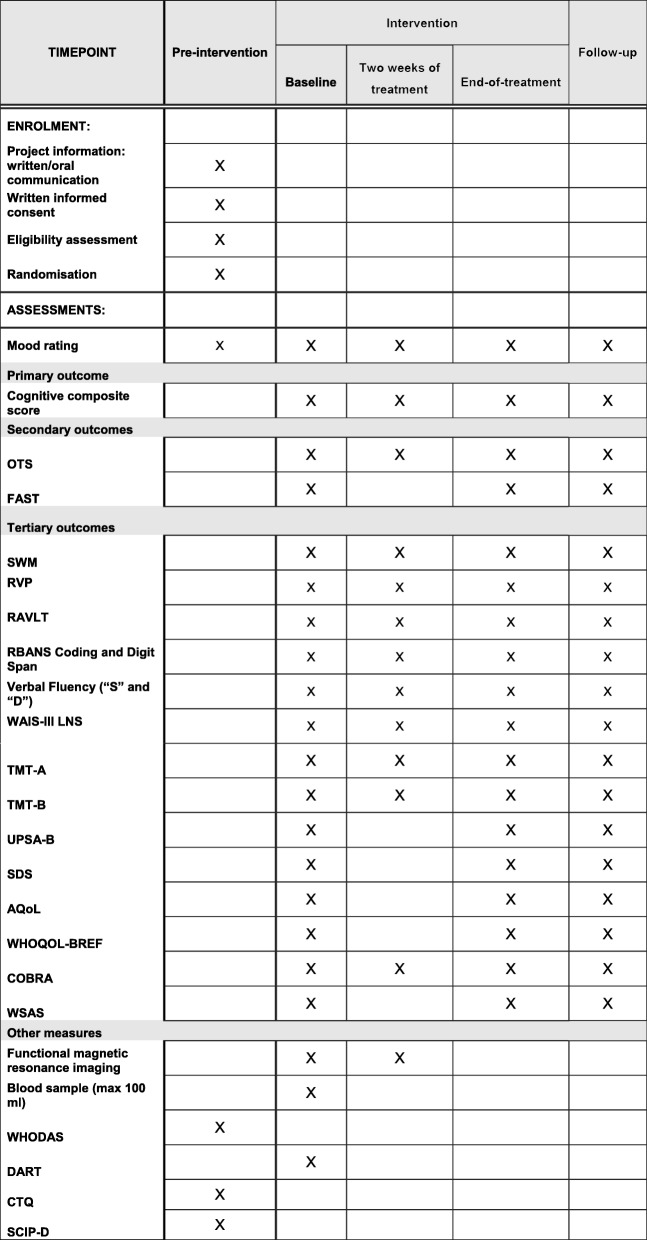
Schedule of enrolment, interventions, and assessments. OTS One Touch Stockings of Cambridge, FAST Functional Assessment Short Test, SWM spatial working memory, RVP Rapid Visual Information Processing, RAVLT Rey Auditory Verbal Learning Test, RBANS Repeatable Battery for the Assessment of Neuropsychological Status, WAIS-III LNS Wechsler Adult Intelligence Scale Version III Letter–Number Sequencing, TMT-A Trail Making Test A, TMT-B Trail Making Test B, UPSA-B Brief Performance-Based Skills Assessment of the University of California, San Diego, SDS Sheehan Disability Scale, AQoL Assessment of Quality of Life, WHOQOL-BREF World Health Organization's Quality of Life Assessment, COBRA Cognitive Complaints in Bipolar Disorder Rating Assessment, WSAS Work and Social Adjustment Scale, WHODAS World Health Organization Disability Assessment Schedule, DART Danish Adult Reading Test, CTQ Child Trauma Questionnaire, SCIP-D Screen for Cognitive Impairment in Psychiatry—Danish version

For an overview of outcome assessment frequency and timing (see Fig. 2). The primary outcome is a broad cognitive composite score, which has been recommended as a primary outcome in cognition trials by the targeting cognition task force of the International Society for Bipolar Disorders (ISBD) [9]. This composite measure comprises the following tests assessing verbal memory, attention, and executive functions: Rey Auditory Verbal Learning Test (RAVLT) [31], Repeatable Battery for the Assessment of Neuropsychological Status (RBANS) Coding [32], verbal fluency with the letter “D” [33], WAIS-III Letter–Number Sequencing [34], Trail Making Test B (TMT B) [35], and Rapid Visual Information Processing (RVP) from the Cambridge Neuropsychological Test Automated Battery (CANTAB) (Cambridge Cognition). The tests in the composite score differ from the exercises included in HappyNeuron Pro. The composite score is derived by averaging the six *z*-transformed test scores.

Secondary cognitive outcome measures are the One Touch Stockings of Cambridge (Cambridge Cognition, CANTAB), which assesses executive functions, and for assessment of functional capacity, the Functional Assessment Short Test (FAST) [36].

The tertiary (explorative) cognitive outcome measures are the RAVLT, RBANS Coding, verbal fluency with the letters “D” and “S”, WAIS-III Letter-Number Sequencing, RBANS Digit Span [32], TMT B and TMT A [35], RVP, and the Spatial Working Memory (Cambridge Cognition, CANTAB) (cognition). Tertiary (explorative) outcomes of objective functional capacity, quality of life, and subjective functioning in daily life are the Brief Performance-Based Skills Assessment of the University of California, San Diego (UPSA-B) [37, 38], the Sheehan Disability Scale (SDS) [39], the Assessment of Quality of Life [40], the World Health Organization's Quality of Life Assessment (WHOQOL-BREF) [41], Cognitive Complaints in Bipolar Disorder Rating Assessment (COBRA) [42], and the Work and Social Adjustment Scale (WSAS) [43].

The cognition outcomes are in line with the latest recommendations from the Targeting Cognition Task Force of the ISBD [44]. Specifically, the recommendations are to include a cognitive composite score as the primary outcome, a single intervention-specific cognition measure as a secondary outcome, and the multiple individual cognition measures as tertiary (exploratory) outcomes. The functional outcomes also agree with the Targeting Cognition Task Force of the ISBD, which recommends FAST or UPSA-B as secondary outcomes. FAST was specified as the secondary functional outcome in this trial since we have previously demonstrated an association between objective cognition and functional capacity measures using it [17]. SDS and WSAS were selected to examine patient-reported outcomes of disability and work function.

#### Peripheral and neural biomarkers and genotype

To assess hypothesis (ii), that early changes in neural activity in the dlPFC may predict pro-cognitive effects of ABCR, participants will undergo fMRI scans at baseline and following 4 weeks of treatment (four active ABCR sessions twice a week or control group sessions twice a week). Blood tests are taken at baseline to investigate any neurobiological predictors of potential pro-cognitive effects of ABCR, including high-sensitivity C-reactive protein (hsCRP), inflammatory and metabolic parameters (lipid status and blood glucose level) [45–49]. These samples will also be used to assess potential differences between genotypes of relevance for cognition, such as the Catechol-O-methyltransferase (*COMT*: val158Met) and Brain-Derived Neurotrophic Factor (*BDNF*: val66Met), relating to treatment-associated improvements in cognition.

### Statistical analyses

Data from the neuropsychological, subjective cognitive impairment, quality of life, level of functioning, and psychosocial function assessments will be analyzed using mixed models. Intention-to-treat analyses will be performed for missing data. The data will be analyzed for every participant for all assessments. No interim analyses will be carried out due to the nature and size of the study.

#### Functional MRI analyses

fMRI data will be preprocessed and analyzed with the FMRIB Expert Analysis Tool (FEAT) and the “randomize” algorithm implemented in the FMRIB Software Library. Neuropsychological and fMRI data from cognitively intact healthy controls without prior or current mental illness or mental illness amongst first-degree relatives from the Bipolar Illness Onset study will be used for baseline comparisons.

#### N-back working memory tasks

fMRI data from the N-back working memory tasks will be analyzed using a region-of-interest analysis to assess differences between the ABCR and control groups in neural activity in the dlPFC after 2 weeks (adjusting for any differences in neural differences at baseline). In addition, we will conduct volume-of-interest analyses of the dorsal prefrontal cortex to examine our hypothesis. Exploratory whole-brain analyses will be conducted to investigate any effects in other brain regions.

#### Strategic picture encoding

We will conduct volume-of-interest analyses of the dorsal prefrontal cortex and the hippocampi to assess the fMRI data from the strategic picture encoding task. Finally, exploratory whole-brain analyses will be conducted to investigate any effects in other brain regions.

Any differences in neural activity will be correlated with potential changes in the cognitive composite score after 2 weeks of treatment and post-treatment. If there is a significant correlation with cognition at post-treatment, multiple regression analysis will be carried out, adjusting for mood and demographic characteristics, to assess whether early changes in neural activity are predictive of pro-cognitive effects.

### Sample size and power calculation

The power calculation was carried out by Pharma Consulting Group (Uppsala, Sweden) (http://www.pharmaconsultinggroup.com) using SAS, based on findings from a previous randomized controlled trial run by our group assessing the effect of 8 weeks of EPO treatment on the same cognitive composite score [50]. The difference between the EPO and saline-treated groups regarding changes on the cognitive composite score was 0.5 standard deviations (SD) from baseline. In this trial, a clinically relevant difference between the ABCR and the control groups following 10 weeks of treatment is assumed to be 0.4 SD (corresponding to a medium effect size) on the primary outcome, with a mean change in the cognitive composite score of 0.5 SD. The power calculation assumes normally distributed data and uses two-sided sample *t-*tests. With these assumptions, we will achieve a power of >80% to detect a clinically relevant difference between the treatment groups at an alpha level of 0.05 with 26 participants in both the ABCR and control groups, respectively. Assuming a 10% drop-out rate from baseline to treatment completion, we will recruit up to 58 participants to achieve complete data sets for 52 participants.

### Data management and monitoring

Personal information is obtained during the eligibility assessment, and information from patient records is accessed only if patients are unable to provide the necessary information. Signed consent forms are kept in a locked filing cabinet. Pseudo-anonymized data from neuropsychological tests, questionnaires, demographic assessments, and interviews will be entered into the Research Electronic Data Capture Database (REDCap). REDCap meets the good clinical practice requirements for data management and storage. The password-protected list matching participant IDs and personal information will be kept separate from the pseudo-anonymized data. Data quality is promoted, as the first author will verify the data entered by the outcome assessors into the REDCap database, and by having range restrictions on values from neuropsychological tests and questionnaires. REDCap features a substantial logging module, which tracks all entered data. The list matching participants IDs and personal information will be deleted and consent forms will be shredded 10 years after study completion, after which the data will be completely anonymized. The entire project group will have access to the final data sets. The Danish Data Protection Agency can conduct inspections to ensure that data management is handled in agreement with the legislation. The Danish Data Protection Agency operates independently of the study. If a participant is excluded or withdraws from the study, the exclusion reason will be recorded, including specification of any adverse events.

## Discussion

The present study investigates the effect of ABCR on cognitive improvement in clinically stable BD patients. It also explores early treatment effects on dorsal prefrontal activity to examine neurocircuitry target engagement. This will contribute to the broader aim of identifying a neurocircuitry biomarker model for pro-cognitive effects.

All participants are required to present with objective cognitive impairment to be enrolled in the study. The criteria are based on findings from our previous EPO trials, showing that patients with worse objective cognitive functioning at baseline have the greatest chance of achieving treatment efficacy [50] and on the subsequent methodological recommendations for cognition trials in BD by the Targeting Cognition Task Force of the ISBD [44]. The criteria will serve to ensure that the sample is enriched for cognitive impairment, which will enhance the chances of detecting treatment-associated cognitive improvement [9]. However, it could cause a recruitment challenge since it is estimated that 30–70% of euthymic patients with BD show clinically relevant objective cognitive impairment [1–3]. As the study will recruit up to 58 patients with BD in full or partial remission to ensure complete data sets for 52 patients, multiple recruitment channels have been identified: psychiatric centers run by Mental Health Services in the Capital Region of Copenhagen, consultant psychiatrists, advertisements, and the Copenhagen Affective Disorder Clinic in Rigshospitalet. A break will interleave the fifth and sixth session in the ABCR program with sessions occurring twice a week, to ensure that all ABCR participants are assessed with fMRI and neuropsychological tests following 2 weeks (four sessions) of treatment.

There are no known direct risks associated with participation in the study. All participants are covered by public insurance provided by the Patient Compensation Association. The use of a control group, where participants are not offered treatment targeting cognitive impairment, will control for non-specific therapeutic effects of being in a group setting, which may increase participants’ quality of life and functional capacity. The use of a control group is also rendered necessary, as there is currently no effective treatment for cognitive impairment with direct and lasting effects [9].

### Trial status and dissemination

A pilot trial was conducted in the autumn and winter of 2016, with minor modifications of some of the practical everyday-like activities to promote feasibility and cultural adaptation. The pilot trial was conducted as a feasibility study and objective cognition was assessed using the SCIP-D rather than a full neuropsychological battery. Objective cognitive impairment was not a requirement. However, three of the five patients included presented with objective cognitive impairment at baseline. All three patients improved numerically on the total SCIP-D score (*M* = 10 and SD = 7), providing some preliminary indications of treatment effect, although the improvement could also be a result of repeated testing given the lack of a control pilot group.

Recruitment will commence in summer 2017 and will complete in the spring of 2019. The final data from the 6-month follow-up will be collected in the autumn/winter of 2019. The results will be disseminated in peer-reviewed scientific journals and at scientific conferences. Author eligibility will be assessed using the Vancouver Convention, and no professional writers will be used. After the follow-up assessment, all participants will receive feedback on their neuropsychological test performance at baseline and after treatment completion.

## Additional file

Additional file 1: SPIRIT 2013 Checklist: Recommended items to address in a clinical trial protocol and related documents. (DOC 122 kb)

## Abbreviations

ABCR: Action-based cognitive remediation
AQoL: Assessment of quality of life
BD: Bipolar disorder
CADIC: Copenhagen Affective Disorder Research Center
COBRA: Cognitive Complaints in Bipolar Disorder Rating Assessment
CTQ: Child Trauma Questionnaire
CR: Cognitive remediation
DART: Danish Adult Reading Test
dlPFC: Dorsolateral prefrontal cortex
EPO: Erythropoietin
FAST: Functional Assessment Short Test
FEAT: FMRIB Expert Analysis Tool
fMRI: Functional magnetic resonance imaging
hsCRP: High-sensitivity C-reactive protein
ISBD: International Society for Bipolar Disorders
OTS: One Touch Stockings of Cambridge
RAVLT: Rey Auditory Verbal Learning Test
RBANS: Repeatable Battery for the Assessment of Neuropsychological Status
REDCap: Research Electronic Data Capture Database
RVP: Rapid Visual Information Processing
SCIP-D: Screen for Cognitive Impairment in Psychiatry—Danish version
SD: Standard deviation
SDS: Sheehan Disability Scale
SWM: Spatial working memory
TMT: Trail making test
UPSA-B: Brief Performance-Based Skills Assessment of the University of California, San Diego
WAIS-III LNS: Wechsler Adult Intelligence Scale Version III Letter–Number Sequencing
WHODAS: World Health Organization Disability Assessment Schedule
WHOQOL: World Health Organization's Quality of Life Assessment
WSAS: Work and Social Adjustment Scale
