# Correction to: Effect of action-based cognitive remediation on cognition and neural activity in bipolar disorder: study protocol for a randomized controlled trial

**DOI:** 10.1186/s13063-019-3289-4

**Published:** 2019-04-08

**Authors:** Caroline V. Ott, Maj Vinberg, Christopher R. Bowie, Ellen Margrethe Christensen, Gitte M. Knudsen, Lars V. Kessing, Kamilla W. Miskowiak

**Affiliations:** 1 Copenhagen Affective Disorder Research Center (CADIC), Psychiatric Centre Copenhagen, Copenhagen University Hospital, Rigshospitalet, Copenhagen, Denmark; 2 Department of Psychology, University of Copenhagen, Copenhagen, Denmark; 3 Psychology Department, Queen’s University, Kingston, ON Canada; 4 Neurobiology Research Unit and Center for Experimental Medicine Neuropharmacology, Rigshospitalet, Copenhagen, Denmark; 5 Faculty of Health and Medical Sciences, University of Copenhagen, Copenhagen, Denmark

**Correction to: Trials (2018) 19:487**

**https://doi.org/10.1186/s13063-018-2860-8**

Following publication of the original article [1], the authors notified us that a comment in the **Peripheral and neural biomarkers and genotype section** was incorrectly phrased during editing. “4 weeks of treatment (four active ABCR sessions twice a week or control group sessions twice a week)” should have actually been described as “two weeks of treatment (4 active, twice a week, ABCR sessions or 2 weekly control group sessions)”.

## References

[1] Ott CV (2018). Effect of action-based cognitive remediation on cognition and neural activity in bipolar disorder: study protocol for a randomized controlled trial. Trials.

